# Risk factors of premature rupture of membranes in public hospitals at Mekele city, Tigray, a case control study

**DOI:** 10.1186/s12884-018-2016-6

**Published:** 2018-09-29

**Authors:** Natnael Etsay Assefa, Hailemariam Berhe, Fiseha Girma, Kidanemariam Berhe, Yodit Zewdie Berhe, Gidiom Gebrehet, Weldu Mamu Werid, Almaz Berhe, Hagos B Rufae, Guesh Welu

**Affiliations:** Adigrat University College of Health Sciences, Adigart, Tigray Ethiopia

**Keywords:** PROM, Risk factors, Case control, Public hospitals, Ethiopia

## Abstract

**Background:**

The incidence of premature rupture of membranes ranges from about 5% to 10% of all deliveries. A woman with premature rupture of membranes is at risk of intra-amniotic infection, postpartum infection, endometritis, and death. A neonate born from premature rupture of membranes mother is at high risk of respiratory distress syndrome, sepsis, intraventricular hemorrhage and death. Little is known regarding the risk factors in Ethiopia. Therefore, this study was conducted to identify risk factors of premature rupture of membranes among pregnant women admitted to public hospitals in Mekelle city, Tigray, Ethiopia.

**Methods:**

Hospital based unmatched case control study design was implemented on 240 samples (160 controls and 80 cases) from pregnant mothers admitted to public hospitals in Mekelle city from February – April/2016. Data was collected by interviewer administered Structured questionnaire and checklist. Binary logistic regression model was used to see the association between dependent and independent variables and multivariable logistic regression was used to identify the independent predictors of premature rupture of membranes.

**Results:**

A total of 160 controls and 80 cases were enrolled in the study. Multivariable logistic regression showed that history of abortion [AOR 3.06 (CI: 1.39, 6.71)], history of PROM [AOR 4.45 (CI: 1.87, 10.6)], history of caesarean section [AOR 3.15(CI: 1.05, 9.46)] and abnormal vaginal discharge in the index pregnancy [AOR 3.31(CI: 1.67, 6.56)] were positively associated with premature rupture of membranes.

**Conclusions:**

Past obstetric history and risks in the index pregnancy have an association with premature rupture of membranes. The finding of the study suggests early identification and treatment of genitourinary infection.

## Background

Premature rupture of the membranes (PROM) is usually defined as rupture of membranes at any time before the onset of uterine contractions. PROM which occurs prior to 37 weeks of gestation is referred as preterm premature rupture of membranes (PPROM), whereas; PROM which occurs after 37 weeks of gestation is referred as term premature rupture of membranes. The latent period is defined as the duration from rupture of the membranes until the onset of true labor [1].

The fetal membrane is composed of the inner amnion and the outer chorion. At term, the amnion is a tough and firm but pliable membrane. This innermost avascular fetal membrane is contiguous with amniotic fluid and occupies a role of incredible importance in human pregnancy. The amnion provides almost all tensile strength of the fetal membranes. Thus, development of its components that protect against its rupture or tearing is vitally important to successful pregnancy outcome [2].

Worldwide, there is a slight difference in the prevalence of premature rupture of membranes and this could be due to the difference in the population studied. The incidence of PROM ranges from about 5% to 10% of all deliveries, and PPROM occurs in approximately 3% of all pregnancies. Approximately 70% of cases of PROM occur in pregnancies at term, but in referral centers, more than 50% of cases may occur in preterm pregnancies. PROM is the cause of about one third of all preterm births [1].

PROM is a significant cause of perinatal morbidity and mortality. The burden of PROM ranges from maternal and neonatal mortality and morbidity to national economic loss due to drug expense, hospitalization, absence from the workplace and expense to the health professionals [3].

Analysis of cost endured by PROM in Mexico showed a total cost for maternity and baby stay of 244,565 dollars and 496,3 97.8 dollars respectively. In addition to other costs, the total cost was 1,029,698.8 dollars. In USA hospital 16,500 dollars was incurred [4, 5].

Premature rupture of membranes results in maternal, infant and neonatal risks. Regarding Maternal risks; Infection of the amniotic cavity is the most common complication after PROM. Endometritis and Abruptio placenta occurs in approximately 2 to 29% and15–25% of cases respectively. Uncommon but serious complications of PROM managed conservatively include retained placenta and hemorrhage requiring dilation and curettage (12%), maternal sepsis (0.8%), and 0.14% maternal death [1, 3].

Fetal complications after membrane rupture include infection and fetal distress due to umbilical cord compression or placental abruption. Because of these factors, women with PROM have a higher risk of cesarean delivery for non-reassuring fetal heart rate. Fetal death occurs in 1 to 2% of cases of conservatively managed PROM. Respiratory distress syndrome (10–40%) is the most common serious acute morbidity after preterm PROM. Necrotizing enterocolitis, and intraventricular hemorrhage are also common. Serious perinatal morbidity can lead to long-term consequences such as chronic lung disease, visual or hearing difficulties, intellectual disabilities, developmental and motor delay, cerebral palsy, or death. Pulmonary hypoplasia is a serious fetal complication which occurs following PROM. (1,3,).

A study conducted in Adis Ababa at Tikur Anbesa Hospital on feto-maternal outcome found the prevalence of PROM to be 1.4%. Intraamnioutic infection was found in 31.5% of the women. Regarding perinatal outcome, 23.6% of them were delivered by c/s, whereas 12 perinatal deaths occurred [6].

So far neonatal and maternal mortality in the nation has declined from 49 per 1000 and 871 per 100,000 in 2000 GC to 28 and 420 in 2014 GC respectively. Despite the achievements, neonatal and maternal mortality is still among the highest in the globe. PROM is one of the major causes of prematurity and infection, which in turn are the leading cause of neonatal death. Consequences of PROM; preterm labour, Low birth weight and neonatal infection are common and significant health problem in Ethiopia [1, 7, 8].

In Ethiopia training manuals and guidelines like Basic Emergency Obstetric & Newborn Care were prepared to make health professionals competent in managing a woman with obstetric emergencies including PROM. Other Strategies were also developed to address the problem including referring women with pre-term prolonged rupture of membranes (longer than 12 h) to a referral-level facility for assessment and Use of prophylactic antibiotics and steroid following pre-labour rupture of membranes [9].

Prediction and prevention of PROM would offer the best opportunity to prevent its complications. The risk factors of PROM include prior preterm birth, cigarette smoking, polyhydramnios, urinary and sexually transmitted infection, prior PROM, work during pregnancy, low Body Mass Index, bleeding, low socioeconomic status [3].

Previous studies to identify the risk factors of PROM were done at different country and different time. Even though, a study was carried out to assess the feto-maternal outcome of PROM, information about the risk factors of PROM in Ethiopia is not available. Hence, this study was conducted to fill this gap by identifying the risk factors.

## Methods

The study was conducted in Mekelle city from December/2015 – June/2016. Mekelle is the capital city of Tigray Region. There are three public hospitals and eight health centers along with many private health institutions [10].

Hospital based unmatched case control study design was implemented. The Study population was pregnant mothers who came to labour ward at public hospitals in mekelle city during the data collection period. Pregnant Women with PROM and without PROM beyond 28 weeks of gestation were included in this study. In Ethiopia, Rupture of membrane before 28 weeks gestation is regarded as abortion.

EPI INFO version 3.5.1 statistical software was used to calculate the sample size using the Double population formula for unmatched case control study by considering the proportion of abnormal vaginal discharge in controls 36.8% with Odds Ratio 2.30 in a previous study done in Uganda [11]. With assumption of 95% CI, 80% power, control to case ratio 2:1 the sample size was 228. By adding 5% non-response rate the total sample size becomes 240 (160 control and 80 cases).

There are three public hospitals in Mekele city. The sample size was allocated to the study hospitals proportionally based on a case flow in the preceding three months. Women with premature rupture of membranes who met the inclusion criteria were recruited consequently until the calculated sample size was attained. Women without premature rupture of membranes who met the inclusion criteria and admitted following the cases was selected using simple random sampling technique as controls and interviewed in the study period.

A Structured questionnaire and checklist developed by the Authors after reviewing different literatures was used. Data was collected by three midwifes. The questionnaire was prepared in English, and translated to Tigrigna, and back to English to ensure consistency. The Data collection was supervised. The collected data was checked for completeness, consistency and clarity.

Data was entered using EpiData version 3.1and exported to SPSS version 20 for analysis. Frequency and percentage was used for categorical variable. Median and inter quartile range was computed. Odds ratio with 95% confidence interval was used to measure strength of association. Bivariate logistic regression model was performed to determine the crude association of the independent variables with the dependent variable. Variables with *P*-value < 0.25 at the bivariate logistic regression were exported to multivariable logistic regression model (backward stepwise) to control confounding factors and to see the independent predictors of PROM. Statistical significance was declared at *P* < 0.05. Efforts were made to confirm the fulfillment of major assumption of logistic regression. Absence of multicolinarity was found to be satisfied. Goodness of fit was checked by Hosmer and Lemeshow test and Omnibus test of model coefficients.

## Results

### Socio-economic and demographic factors

Two hundred forty participants (*n* = 80 for cases and *n* = 160 for controls) were enrolled in the study. The median age of the women for the case and control were 27 (IQR = 5) and 26 (IQR = 7) respectively. More than half of cases and controls were housewives, and the majority of cases and controls have attended more than secondary Education. Regarding residence, majority of cases and controls were living in urban. Majority of cases and controls belong to Tigray ethnicity. The proportion of participants earning < 1000 Ethiopian birr (1 us dollar = 27 birr) was higher in the cases than controls. Proportion of partner job, as a daily laborer is higher in cases than controls (Table 1).

**Table 1:** Socio-demographic characteristics of cases and controls attending public hospitals of Mekelle city, Tigray, Ethiopia 2016

VARIABLES	CATEGORY	Cases *N* = 80(%)	Controls *N* = 160(%)	Total *N* = 240(%)
AGE	15–19	1(1.2)	10(6.2)	11(4.6)
	20–34	73(91.2)	132(82.5)	205(85.4)
	35–49	6(7.5)	18(11.2)	24(10)
Educational level	No formal Education	13(16.2)	29(18.1)	42(17.5)
	Primary	18(22.5)	30(18.8)	48(20)
	Secondary	19(23.8)	52(32.5)	71(29.6)
	More than secondary	30(37.5)	49(30.6)	79(32.9)
Mothers occupation	House wife	47(58.8)	90(56.2)	137(57.1)
	Merchant	14(17.5)	27(16.9)	41(17.1)
	Employee	15(18.8)	31(19.4)	46(19.2)
	Others^a^	4(5)	12(7.5)	16(6.7)
Residence	Urban	68(85)	133(83.1)	201(83.8)
	Rural	12(15)	27(16.9)	39(16.2)
Monthly Income^b^	< 1000	18(22.5)	24(15)	42(17.5)
	1001–2000	13(16.2)	39(24.4)	52(21.7)
	2001–3000	18(22.5)	31(19.4)	49(20.4)
	> 3000	31(38.8)	66(41.2)	97(40.4)
Marital status	Unmarried	4(5)	11(6.9)	15(6.2)
	Married	76(95)	149(93.1)	225(93.8)
Ethnicity	Tigray	76(95)	153(95.6)	229(95.4)
	Non Tigray	4(5)	7(4.4)	119(4.6)
Partner Educational level	No formal education	12(15)	29(18.1)	41(17.1)
	Primary	9(11.2)	29(18.1)	38(15.8)
	Secondary	22(27.5)	39(24.4)	61(25.4)
	More than secondary	37(46.2)	63(39.4)	100(41.7)
Partner job	daily laborer^***^	11(13.8)	21(13.1)	32(13.3)
	merchant	18(22.5)	30(18.8)	48(20.0)
	employee	44(55)	89(55.6)	133(55.4)
	others^a^	7(8.8)	20(12.5)	27(11.2)

^a^ E.g. farmer, student

^b^ = Ethiopian Birr

***Daily laborer- Daily laborer means someone who doesn’t have a formal training, profession or employment, and does whatever job comes its way with a minimum and daily wage for a living

### Physical activity habits

The majority of the participants have no history of carrying heavy objects. Regarding to sleep more than two third of cases and controls reported that they sleep less than 8 h per day. Two in ten of women in cases and 15% of controls use transportation daily. It was observed that low proportion of cases and controls have a maid (Table 2).

**Table 2:** Distribution of Risky physical activity habits among women in the case and control groups attending Public Hospitals of mekelle city, Tigray Ethiopia, 2016

Variable	Category	PROM status		Total
		Case *n* = 80(%)	Control *n* = 160(%)	
Carry heavy objects	Yes	28(35)	34(21.2)	62(25.8)
Sleep < 8 hours	Yes	25(31.2)	35(21.9)	60(25)
Daily transportation	Yes	17(21.2)	24(15)	41(17.1)
Help at home	I don’t have	20(25)	61(38.1)	81(33.8)
	My husband	18(22.5)	20(12.5)	38(15.8)
	Maid	16(20)	41(25.6)	57(23.8)
	Family	26(32.5)	38(23.8)	64(26.7)

### Past obstetric and gynecologic history

Twenty-five (31.2%) of cases and 10.6% of controls had an abortion. Among the women who had an abortion, almost quarter of cases and 1 out of 10 controls had two abortions. One hundred two controls and 77.8% of cases have no history of preterm delivery. With regard to PROM one third of pregnant women in the case group and a small number of pregnant women in the control group have history of PROM. A few women in both case and control groups had cervical operation and cerclage. More than half of pregnant women, in case group and almost two third of pregnant women in control group has used modern contraceptive; the injectable being the commonly used one. Lower proportion of women had caesarean section in both case and control groups (Table 3).

**Table 3:** Past obstetric and gynecologic history of women among cases and controls attending public hospitals of mekelle city, Tigray Ethiopia

Variable	Category	PROM status		Total
		Case *n* = 80(%)	Control *n* = 160(%)	
Abortion	Yes	25(31.2)	17(10.6)	42(17.5)
Number of Abortion	1	17(68)	15(88.2)	32(76.2)
	2	6(24.0)	2(11.8)	8(19)
	3	2(8)	0(0)	2(4.8)
Type of Abortion	Spontaneous	18(72)	13(76.5)	31(73.8)
	Induced	5(20)	4(23.5)	9(21.49)
	Both	2(8)	0(0)	2(4.8)
Preterm delivery	Yes	6(7.5)	18(11.2)	24(10)
Previous PROM	Yes	27(33.8)	10(6.2)	37(15.4)
Cervix operation	Yes	4(5)	3(1.9)	7(2.9)
Cervical cerclage	Yes	3(3.8)	4(2.5)	7(2.9)
Modern contraceptive	Yes	48(60)	101(63.1)	149(62.1)
Contraceptive type	IUCD	2(4.2)	4(4)	6(4)
	Pill	7(14.6)	19(18.8)	26(17.4)
	Injectable	36(75)	64(63.4)	100(67.1)
	Implant	3(6.2)	14(13.9)	17(11.4)
C/S	Yes	13(16.2)	7(4.4)	20(8.3)

### Risks in the index pregnancy

The median gestational age of the pregnant women was 37.25 (IQR = 3.4) and 38.4(IQR = 2.8) for cases and controls respectively. Proportions of Primigravida and Nullipara mothers were higher in the control group than cases. A small number of cases and controls had mid-upper arm circumference below 23 cm. Abnormal vaginal discharge was reported by almost half of cases and 15.6% controls. The proportion of mothers who had antenatal care follow up in the cases, and controls was high. More than half of cases and controls had intercourse during third trimester. With regard to smoking, the number of active and passive smokers was few. The proportion of women who had vaginal bleeding in case and control group was low. The majority of cases and controls had no history of accident. Majority of women in both case and control groups had cephalic presentation. Number of PIH, Polyhydramnious and Multiple pregnancies were very few in both groups (Table 4a and b).

**Table 4:** Risks in the index pregnancy of women among cases and controls attending public hospitals of mekelle city, Tigray Ethiopia

Variable	Category	PROM status		Total
		Case *n* = 80(%)	Control *n* = 160(%)	
GA	< 37	33(42.3)	18(11.8)	51(22.2)
	37–42	42(53.8)	128(84.2)	170(73.9)
	> 42	3(3.8)	6(3.9)	9(3.9)
Gravidity	1	19(23.8)	60(37.5)	79(32.9)
	1–4	50(62.5)	84(52.5)	134(55.8)
	> 5	11(13.8)	16(10)	27(11.2)
Parity	0	25(31.2)	65(40.6)	90(37.5)
	1	28(35)	51(31.9)	79(32.9)
	2–4	22(27.5)	34(21.2)	56(23.3)
	> 5	5(6.2)	10(6.2)	15(6.2)
Hemoglobin level	< 11	4(5.1)	4(2.5)	8(3.4)
	> 11	75(94.9)	155(97.5)	230(96.6)
MUAC	< 23	11(13.8)	24(15)	35(14.6)
	> 23	69(86.2)	136(85)	205(85.4)
Abnormal Vaginal discharge	Yes	39(48.8)	25(15.6)	64(26.7)
ANC	Yes	78(97.5)	158(98.8)	236(98.3)
Number of Visit	1	1(1.3)	6(3.8)	7(3)
	2	1(1.3)	3(1.9)	4(1.7)
	> 2	76(97.5)	149(94.3)	225(95.3)
Chronic cough	Yes	28(35)	44(27.5)	72(30)
Third trimester intercourse	Yes	49(61.2)	84(52.5)	133(55.4)
Smoking/active or passive	yes	8(10)	13(8.1)	21(8.8)
Coffee intake	No	17(21.2)	8(5)	25(10.4)
	< two cup	32(40)	72(45)	104(43.3)
	>two cup	31(38.8)	80(50)	111(46.2)
Vaginal bleeding	Yes	7(8.8)	9(5.6)	16(6.7)
Accident	No	73(91.2)	151(94.4)	224(93.3)
	Fall	7(8.8)	9(5.6)	16(6.7)
Alcohol	Yes	24(30)	24(15)	48(20)
Presentation	Cephalic	71(88.8)	150(93.8)	221(92.1)
	Breech	8(10)	8(5)	16(6.7)
	Others	1(1.2)	2(1.2)	3(1.2)
PIH	Yes	5(6.2)	5(3.1)	10(4.2)
Polyhdramnious	Yes	1(1.2)	0(0)	1(0.4)
Multiple pregnancy	Yes	2(2.5)	4(2.5)	6(2.5)

### Risk factors of premature rupture of membranes (bivariate and multivariate analysis)

The bivariate analysis showed that only 13 variables were with a P- value less than 0.25. The 13 variables with a P- value less than 0.25 have been exported to the multivariable logistic regression. The multivariable analysis showed that only four variables have shown a significant association.

Accordingly, History of Abortion was found significantly associated with PROM. Those who had abortion were 3.06 times more likely to develop premature rupture of membranes with AOR 3.06 (CI: 1.39, 6.71). History of C/S also showed a significant association with the AOR 3.15(CI: 1.05, 9.46). Previous premature rupture of membrane was the strongly associated risk factor than the others. The odds of developing premature rupture of membranes among women who had previous PROM was 4.45 times higher than who had not history of PROM. Furthermore, abnormal vaginal discharge in the index pregnancy was also significantly associated risk factor with AOR 3.31(CI: 1.67, 6.56). Whereas, carrying heavy objects, monthly income, sleep < 8, smoking, chronic cough, sexual intercourse, gravidity and parity, and accident were not associated with PROM (Table 5).

**Table 5:** Bivariate and multivariable logistic regression (final model) analysis result for significant variables (*p* ≤ 0.25) in bivariate analysis in Mekelle City Public Hospitals, North Ethiopia, 2015

Variable	Category	COR (95% CI)	AOR (95% CI)
Monthly income	< 1000	1	1
	1001–2000	0.44(0.18,1.07)	0.37(0.13, 1.05)
	2001–3000	0.77(0.33, 1.80)	0.57(0.21, 1.53)
	> 3000	0.63(0.30, 1.32)	0.63(0.26, 1.52)
Carrying heavy objects	No	1	1
	Yes	1.99(1.10, 3.62)^a^	1.63(0.81, 3.30)
Sleep < 8	No	1	1
	Yes	1.62(0.89, 2.97)	1.25(0.59, 2.64)
Abortion	No	1	1
	Yes	3.82(1.91, 7.62)^a^	3.06 (1.39, 6.71)^a^
Previous PROM	No	1	1
	Yes	7.64(3.47, 16.8)^a^	4.45 (1.87, 10.6)^a^
CS	No	1	1
	Yes	4.24(1.62, 11.1)^a^	3.15(1.05, 9.46)^a^
Abnormal Vaginal discharge	No	1	1
	Yes	5.13(2.78, 9.47)^a^	3.31(1.67, 6.56)^a^
Chronic cough	No	1	1
	Yes	1.42(0.80, 2.53)	0.75(0.36, 1.56)
Third trimester sexual intercourse	No	1	1
	Yes	1.43(0.83, 2.47)	1.44(0.75, 2.76)
Alcohol Intake	No	1	1
	Yes	2.42(1.27, 4.63) ^a^	1.93(0.91, 4.08)
History of accident	No	1	1
	Yes	4.33(1.26,14.8) ^a^	2.27(0.69, 7.40)
Gravidity	1	1	1
	1–4	1.88(1.01, 3.51) ^a^	1.80(0.30,10.6)
	> 5	2.17(0.86, 5.48)	2.76(0.20, 36.8)
Parity	0	1	1
	1	1.48(0.77, 2.82)	0.66(0.29, 1.48)
	2–4	1.60(0.78,3.28)	0.50(0.19, 1.32)
	> 5	1.30(0.40, 4.18)	0.29(0.06, 1.45)

*COR* = Crude odds ratio, *AOR* = adjusted odds ratio

^a^Significant association

## Discussion

The aim of this unmatched case control study was to identify the risk factors of premature rupture of membranes. This study identified that history of abortion, history of C/S, previous PROM and abnormal vaginal discharge to be significantly associated with PROM.

In our study, socio-demographic factors have no significant association with the occurrence of premature rupture of membranes though it was observed that a higher proportion of women below 1000 Ethiopian Birr (45 US dollar) per month in case group than the control. The result is in discordant with findings from Canada, USA, Brazil, Lithuania, Australia, Pakistan and India [12–18]. However, the finding is in line with researches done in Egypt and Uganda [19, 20]. The reason might be due to the participants sharing similar background.

In this study history of abortion was found to be a risk factor for PROM. Participants who had a history of abortion were 3.06 times more likely to develop PROM than who had no abortion. The number and type of abortion were not significant. This is supported by researches done in USA, Lithuania, India, China and Uganda [18, 19, 15, 21, 22]. Unlike our study Kaya in Uganda found a significant association between two and more induced abortions and PROM [19]. This difference might be due to low prevalence of induced abortion in our study.

Literatures from USA and China revealed that history of preterm birth to be a risk factor for premature rupture of membranes [21, 22]. This study however revealed no significant association between history of preterm birth and premature rupture of membranes. This is in line with other researchers from Lithuania, India, Pakistan and Uganda [15, 17–19].

Several studies from USA, Sweden, India, Thailand, Egypt, Nigeria and Uganda revealed that previous PROM was a significant risk factor for premature rupture of membranes [18–21, 23]. This study also showed that previous PROM to be the strongest risk factor for premature ruptures of membranes. Women who had previous PROM were 4.45 more likely to develop PROM with AOR 4.45 (CI: 1.87, 10.6). This might be due to untreated genitourinary infection and a short cervical length. In addition, obstetric problems are recurrent by nature.

Cervical cerclage was found to be a risk factor for PROM in the literatures of India and Uganda. This study failed to detect a significant association between a cervical cerclage and PROM since the factor was low in both groups [18, 19]. Similarly, our study revealed no significant association between history of operation on the cervix and PROM. This is supported by a study from India and Uganda [18, 19].

A study from Uganda revealed that caesarean section was a significant risk factor for premature rupture of membranes [19]. Our study also found the caesarean section to be a significant risk factor. Participants with history of C/S were 3.15 times more likely to develop PROM than who hadn’t history of c/s. This might be due to the increased risk of rupture of C/S scar in the subsequent pregnancy.

Abnormal vaginal discharge has shown a significant association with the occurrence of PROM. Women who had an abnormal vaginal discharge in the index pregnancy were 3.31 times more likely to develop PROM. This is in line with a study conducted in Uganda and Egypt [19, 20]. Abnormal vaginal discharge is indicative of infection. Infection causes inflammation of the membranes leading to the rupture. Some genital bacteria elaborate enzymes such as proteases, phospholipases, and collagenases which cause membrane weakness and rupture [1].

In a study conducted in India and Uganda Antenatal care less than two follow up was found associated with PROM. During ANC visit there is a period for counseling and health Education. This component helps pregnant women to keep their personal hygiene and avoid risky habits. In our study ANC was not found significantly associated with PROM. The reason might be ANC coverage in both groups was similarly high. Therefore, the study couldn’t identify a significant difference [18, 19].

Several researchers found sexual intercourse during late pregnancy to be a risk factor to develop PROM. However, in this research association was not found between sexual intercourse and PROM. This is supported by a finding from Egypt [18–20, 24].

Nicotine, which is found in cigarette, causes constriction of arteries leading to ischemia of the decidua. Studies done in USA, India and Uganda revealed that smoking increases the risk of developing premature rupture of membranes. In this study there was no significant association found between smoking and PROM. This might be due to the low prevalence of the smoking in the study participants. Similarly, chronic cough and alcohol intake were not significant. This is comparable with a study done in Nigeria and Uganda [18–21, 24].

Vaginal bleeding, Accident, gestational age, Gravidity, parity, presentation, Polyhydramnious, multiple pregnancy, Anemia and Pregnancy Induced Hypertension were not found to be a significant predictor of premature rupture of membranes. This is discordant with findings from a research conducted in Sweden, Lithuania and India [18, 19, 15, 25].

## Conclusion

In conclusion, this study found that past obstetric history and factors in the index pregnancy have association with premature rupture of membranes. The study revealed history of abortion, previous premature rupture of membranes, history of caesarean section and abnormal vaginal discharge in the index pregnancy to be a risk factor for premature rupture of membranes.

## Abbreviations

ANC: Ante-natal care
IUCD: Intra uterine contraceptive device
MDGs: Millennium development Goals
OR: Odds ratio
PPROM: Preterm premature rupture of membranes
PROM: Premature rupture of membranes

## References

[1] Gibbs R, Karlan B, Haney A, Nygaard I. Danforth’s obstetrics and gynecology. 10th ed. Philadelphia: Lippincott Williams & Wilkins; 2008.

[2] Cunnigham FGLK, Bloom SL, Hauth JC. Williams’s obstetrics. 23th ed. USA: McGraw-Hill Companies; 2010.

[3] Gabbe SG, Niebyl JR, Simpson JL. Obstetrics: Normal and problem pregnancies. 5th ed: Ed: Churchill Livingstone; 2007.

[4] Nava Flores JEMM, Hernández-Valencia M. Maternal and fetal morbidity in patients with premature rupture of the membrane after 27-week gestation. Causes and Costs. 14515665

[5] Wanda NKF, Neil P (2000). Economic burden of hospitalizations for preterm labor in the United States. American College of obstetricians and Gynecologists.

[6] Sirak B, Mesfin E. Maternal and perinatal outcome of pregnancies with PROM at Tikur Anbesa specialized teaching hospital. Ehtiop Med J. 2014;(4):52. 26410989

[7] Ministry of Finance and Economics (2012). Ethiopia MDGs report.

[8] Agency ECS, ICF International (2014). Ethiopia demographic and health survey. Addis Ababa E, and Calverton.

[9] Addise M. Maternal and Child Health Care: Lecture note: University of Gondar; 2003.

[10] Agency FDRoECS (2013). Population Projection of Ethiopia for All Regions at Wereda Level from 2014–2017.

[11] Sarah N, Dan K, Freddie B, Nazarius M, Florence M. Genital infections and risk of premature rupture of membranes in Mulago Hospital,Uganda: a case control study. BMC Res Notes. 2015. 10.1186/s13104-015-1545-6 PMC4608222 26475265

[12] Fergusun SE, Smith GN, Salenieks ME, Windrim R, Walker MC (2002). Preterm premature rupture of membranes; nutritional and socio demographic factors. Obstet Gynecol.

[13] A S, Nicola S, Piazzi G, Ghazal K, Colonna L, Baltaro F (1994). Epidemiological correlates of preterm premature rupture of membranes. Int J Gynaecol Obstet.

[14] Arnildo A, Elaine P, Tania M. Preterm premature rupture of the fetal membranes: association with socio-demographic factors and maternal genitourinary infections. J De Pediatrica. 2014;90(2). 10.1016/j.jped.2013.08.003 24184300

[15] Daiva V, Stafan B, Vidaa M. Antenatal risk factors associated with PPROM. Acta Medica Litutania. 2002;9(3).

[16] Sarah J. Demographic, clinical and environmental risk factors for PROM in western Australia: University of western Australia; 2008.

[17] Shehla N, Ali F, Rubina B, Ruqqia S. Prevalance of PPROM and its outcome. J Ayub Med College. 19(4):15–6. 18693588

[18] Choudhary M, Rathore SB, Chowdhary J, Garg S. Pre and post conception risk factors in PROM. Int J Res Med Sci. 2015;3(10).

[19] Kaye D (2001). Risk factors for preterm premature rupture of membranes at Mulago Kampala Uganda. East Afr Med J.

[20] Tarek KA, Sahar NM, Hamida AE, Amal AA, et al. Cervicovaginal Infection during Pregnancy and Its Relation to Preterm Pre-Labour Rupture Of Membranes. J Am Sci. 2012;8(12).

[21] Edem E, Carol A, Robert W, Atef M. Risks for premature rupture of amniotic membranes. Int J Epidemiol. 1993;22(3). 10.1093/ije/22.3.495 8359967

[22] Zhou Q, Zhang W, Xu H, Liang H, Ruan Y, Zhou S (2014). Risk factors for preterm premature rupture of membranes in Chinese women from urban cities. Int J Gynecol Obstet.

[23] Ekachai K, Patipan S. Risk factors related to premature rupture of membranes in term pregnant womens. Aust N Z J Obstet Gynaecol Zoao. 2000;40(1). 10.1111/j.1479-828x.2000.tb03162.x 10870775

[24] Emechebe CI, Njoku CO,KA, Udofia U (2015). Determinants and complications of pre-labour rupture of membranes (PROM) at the University of Calabar Teaching Hospital (UCTH), Calabar, Nigeria. Scholars J Applied Med Sci.

[25] Lars L (1998). Clinical and epidemiological studies: premature rupture of membranes. Br J Obstet Gynaecol.

