# Correction to: Risk factors of premature rupture of membranes in public hospitals at Mekele city, Tigray, a case control study

**DOI:** 10.1186/s12884-018-2042-4

**Published:** 2018-10-16

**Authors:** Natnael Etsay Assefa, Hailemariam Berhe, Fiseha Girma, Kidanemariam Berhe, Yodit Zewdie Berhe, Gidiom Gebreheat, Weldu Mamu Werid, Almaz Berhe, Hagos B Rufae, Guesh Welu

**Affiliations:** Adigrat University College of Health Sciences, Adigart, Tigray Ethiopia

## Correction

Following publication of the original article [1], the author reported that his name was misspelled. The original article has been corrected.

Incorrect name: Gidiom Gebrehet

Correct name: Gdiom Gebreheat
