# Correction to: Risk factors of premature rupture of membranes in public hospitals at Mekele city, Tigray, a case control study

**DOI:** 10.1186/s12884-020-2723-7

**Published:** 2020-01-13

**Authors:** Natnael Etsay Assefa, Hailemariam Berhe, Fiseha Girma, Kidanemaryam Berhe, Yodit Zewdie Berhe, Gdiom Gebreheat, Weldu Mamu Werid, Almaz Berhe, Hagos B. Rufae, Guesh Welu

**Affiliations:** Adigrat University College of Health Sciences, Adigart, Tigray Ethiopia

**Correction to: BMC Pregnancy Childbirth**

**https://doi.org/10.1186/s12884-018-2016-6**

Following publication of the original article [1], we have been notified that the name of one author was spelled incorrectly as Kidanemariam Berhe, when the correct spelling is Kidanemaryam Berhe.
